# A High-Throughput and Robust Relative Potency Assay Measuring Human Cytomegalovirus Infection in Epithelial Cells for Vaccine Development

**DOI:** 10.3390/vaccines13060626

**Published:** 2025-06-10

**Authors:** Nicole M. Smiddy, Nisarg Patel, Matthew C. Troutman, Kristine M. Kearns, Zachary P. Davis, Christopher S. Adams, Carl Hofmann, Donald J. Warakomski, Harrison Davis, Daniel Spatafore, Adam Kristopeit, Pete DePhillips, John W. Loughney

**Affiliations:** 1 Analytical Research and Development, Merck & Co., Inc., Rahway, NJ 07065, USA; matt.troutman83@gmail.com (M.C.T.); kristine.kearns@optimizeyourlab.com (K.M.K.); zpdavis93@gmail.com (Z.P.D.); chris.adams1@merck.com (C.S.A.); carl.hofmann@merck.com (C.H.); donald_warakomski@merck.com (D.J.W.); pdsld@comcast.net (P.D.); john_loughney@merck.com (J.W.L.); 2 Vaccine Drug Product Development, Merck & Co., Inc., Rahway, NJ 07065, USA; harrison_davis@merck.com; 3 Process Research and Development, Merck & Co., Inc., Rahway, NJ 07065, USA; daniel.spatafore@merck.com (D.S.); adam_kristopeit@merck.com (A.K.)

**Keywords:** cytomegalovirus, vaccine, potency, infectivity, automation

## Abstract

**Background/Objectives:** A preventative vaccine against human cytomegalovirus (HCMV) infection and disease remains an unmet medical need. Several attenuated virus and antigen-based HCMV vaccine candidates have been proposed; however, development challenges have limited their progression through the clinical pipeline. **Method:** A high-throughput and robust relative potency assay, Imaging of Relative Viral Expression (IRVE), was developed and applied to measure the infection of a live-attenuated HCMV vaccine candidate in ARPE-19 epithelial cells. The IRVE assay measures HCMV infection by immunostaining Immediate Early 1 (IE1) protein and enumeration of IE1-positive, infected cells against total cells. Increased throughput was accomplished using 384-well plate automation on a custom-designed integrated robotic system. **Results:** The IRVE assay effectively measures relative potency changes in an HCMV vaccine candidate under different upstream processes, downstream processes, and formulation conditions. Key assay parameters including microplate format, cell density, serum concentration, infection time and influence of cell age were evaluated and optimized. The IRVE assay was correlated to historical, lower throughput HCMV potency assays, including plaque and Infectivity of Early Gene Expression (IEE), validating its application as a potency screening tool. **Conclusions:** The IRVE assay has been successfully implemented to support HCMV vaccine development over several years of clinical development.

## 1. Introduction

Human herpesvirus 5, more commonly known as human cytomegalovirus (HCMV), is among the largest viruses known to cause disease in humans. HCMV is an enveloped, DNA virus comprising a 235–250 kb linear double-stranded genome encoding an estimated 162–252 open reading frames, many of which are essential for viral replication in host cells such as fibroblasts and epithelial cells [1]. HCMV infection and its varying pathways to disease continue to present an unmet medical need, particularly in more vulnerable populations. Infection with HCMV can cause mild disease in immunocompetent children and adults, while more severe disease is observed in immunocompromised persons, including those receiving cancer therapy, solid-organ transplant patients, and seronegative women who may contribute to congenital disease. Congenital HCMV infection is reported to impact 0.5–2% of all pregnancies each year and is proposed to be a main cause of congenital hearing loss and neurologic damage [2].

A vaccine against HCMV for the prevention of newly acquired infections and latent virus reactivation could reduce or eliminate HCMV-associated disease burden and mortality [3,4,5]. HCMV vaccine development strategies include virus attenuation to produce a modified virus vaccine, as well as isolation of subunit viral antigens to create individual antigen vaccines [6]. Despite HCMV vaccine development efforts, no licensed vaccine is currently available. Failure to induce neutralizing antibody titers comparable to those with naturally acquired immunity is a limitation of many proposed vaccine candidates [7]. Immunogenicity challenges are influenced by HCMV genetic and structural complexity, and sophisticated mechanisms for host cell entry, immune evasion, and persistence [1].

A deeper understanding of HCMV pathogenesis is enabling advancements in vaccine strategies [1,6]. Improvements in live-attenuated vaccine strategies, specifically, are valuable in that the strains mimic native viral infection in the host cells and elicit both humoral and cellular immune response while causing only mild disease with few, if any, symptoms [8,9]. For instance, the decreased immunogenicity of many HCMV vaccine strains results from a missing pentameric glycoprotein H (gH) complex known to be critical for receptor-mediated viral entry of endothelial/epithelial cells and leukocytes [7,10]. In response to this discovery, a live-attenuated vaccine construct was proposed comprising a restored pentameric gH complex and demonstrated improved specificity for epithelial cells [11,12]. This construct was also designed with a genetic, chemical switch to be conditionally replication-defective, thus reducing viral pathogenesis by permitting a single replication cycle post-infection while blocking the production of subsequent viral progeny unless in the presence of the synthetic compound, Shield-1.

For successful HCMV vaccine development, a robust in vitro potency assay is required to study the impact of vaccine process and formulation on potency. Potency assays are implemented throughout a vaccine’s life cycle to ensure it contains the appropriate biochemical properties to elicit the required immune response. Live-attenuated vaccine potency is determined by the virus’s ability to replicate (i.e., infectivity) and its dose (i.e., number of viral particles delivered) which are often measured using infectivity and relative potency assays, with plaque assays serving as the industry standard. When developing a potency assay, understanding the virus’s mechanism of action helps to inform many key parameters, including the selection of the host cells, viral antigen measured, and infection incubation time [13]. For HCMV, virus attachment and entry into host cells is achieved via cell type-specific protein interactions and the resulting infection causes a temporally regulated cascade of gene expression [14,15]. One of the first proteins expressed after HCMV infection is immediate-early 1 (IE1) protein which can be directly measured to monitor viral infection [16]. After infection, HCMV exhibits a uniquely slow eclipse time of four to five days when the virus replicates in the nucleus of single cells without causing secondary, satellite infections of other cells. HCMVs slow replication time permits a full capture of viral particle concentration relative to the inoculation fluid volume and number of host cells infected. New HCMV virions are eventually trafficked to the plasma membrane and outside of the cell to infect other host cells [14,15].

The majority of HCMV potency assays are limited in throughput due to the intrinsically slow growth of HCMV, manual labor involved, and low sample capacity. Traditional infectivity assays such as tissue culture infectious dose 50 (TCID_50_) and plaque assays measure cell death or plaque formation, respectively, usually requiring several weeks to elicit the measurable response and suffering from poor reproducibility and/or low sensitivity. HCMV potency assays with improved sensitivity and/or processing time include quantitative polymerase chain reaction, DNA hybridization, branched-DNA signal amplification and shell vial centrifugation culture assays; however, these methods remain rather time-consuming or not readily adaptable to high-throughput screening [17]. Inherent variability in the potency assays described may also be addressed by measuring relative instead of absolute potency. Relative potency can be measured by evaluating a reference standard and test sample dilution series in parallel whereby the half maximal effective dilution (ED_50_) value (e.g., dilution at which 50% of the cells are infected) for each sample is determined and the ratio of the reference ED_50_ to the test sample ED_50_ calculated [13]. Relative potency assays measuring viral antigens have been used to enable the detection of infection at shorter infection times (e.g., ~1–2 days) via, for example, IE1 immunostaining [18] and fluorescent fusion proteins [19].

While assay options exist to shorten turnaround time for results, manual sample handling remains a limiting factor in assay throughput. To increase the throughput of the HCMV vaccine process and formulation screening and streamline vaccine development efforts, the introduction of assay miniaturization and automation is necessary. Additionally, it is desirable to be able to test all samples from a single study in the same assay run to eliminate any discrepancies between sample results due to inherent assay variability (e.g., different upstream bioreactor conditions, downstream process intermediates, and formulation stability time points). Such assay miniaturization and automation have been accomplished for other viral potency methods, including plaque assays for Dengue virus [20] and relative potency assays for Varicella Zoster Virus [21].

To address existing HCMV potency method challenges and streamline live-attenuated HCMV vaccine development, the high-throughput and robust relative potency assay Imaging of Relative Viral Expression (IRVE) was developed. The automated 384-well plate IRVE assay measures HCMV infection in ARPE-19 epithelial cells by counting cell nuclei from microscopy images using a nuclear stain and the corresponding IE1-positive HCMV-infected nuclei via immunofluorescence. Key assay variables were evaluated during development to promote assay robustness, including microwell plate format, cell density, serum concentration, infection time, viral concentration, and cell passage number. This method proves superior in infection time, precision, and throughput compared to traditional and previously reported methods measuring HCMV infectious virions. Since the development of the IRVE assay, its methods have been used to support numerous HCMV research and vaccine development studies, some of which have been previously reported in the literature [22,23,24]. However, details related to the assay development and performance have not been previously described nor has the label “IRVE” been used prior. Here, for the first time, the development and application of the IRVE assay for live-attenuated HCMV vaccine process and formulation development is explained.

## 2. Materials and Methods

### 2.1. Cell Maintenance

Maintenance of human spontaneously arising retinal pigment epithelia (ARPE-19) cell line (ATCC, Manassas, VA, USA, CRL-2302) was performed using a CompacT SelecT automated cell culture system (Sartorius Stedim BioTech, Bohemia, NY, USA) equipped with a Cedex cell counter (Roche Diagnostics, Indianapolis, IN, USA). ARPE-19 cells were thawed and seeded manually into T-175 flasks (Corning, Corning, NY, USA, 430106) containing culture medium comprising DMEM/F-12 (Corning, 10-090-CV) with 2% L-Glutamine (Corning, 10-090-CM) supplemented with 10% fetal bovine serum (FBS) (HyClone, Logan, UT, USA, SH30070.03) and 1% Penicillin/Streptomycin (Pen/Strep) (Gibco, Waltham, MA, USA, 15140-122). Cell flasks were passaged four days post-thaw and serially passaged once a week there-after at 8 × 10^5^ cells per flask using TrypLE Express (Invitrogen, Waltham, MA, USA) spiked with 0.01% Pluronic acid (Thermo Scientific, Waltham, MA, USA). Following two passages in T-175 flasks, the cells were transitioned to HYPERflasks (Corning, 10076) at 7.8 × 10^6^ cells per flask for additional cell expansion.

### 2.2. HCMV Samples

Live-attenuated HCMV vaccine construct samples were acquired from Merck & Co., Inc., Rahway, NJ, USA [11,12] at different stages throughout vaccine development, including upstream, downstream, and formulation. Generally, the upstream process propagated HCMV construct in ARPE-19 cells cultured on microcarriers in bioreactors, as previously described [24]. The downstream process involved various chromatography and buffer exchange steps to purify and concentrate the HCMV construct [25]. The resulting drug substance (DS) was flash-frozen and stored at −70 °C. The DS was thawed and formulated into a liquid matrix comprising various excipients to produce the drug product (DP) which was stored as a liquid or as a lyophilized cake at −70 °C. Proprietary sample details are omitted.

### 2.3. Cell Plating and Infection

ARPE-19 cells were harvested and suspended in plant medium comprising DMEM/F-12 (Corning, 10-090-CV) with 2% L-Glutamine (Corning, 10-090-CM) supplemented with 1% FBS (HyClone, SH30070.03) and 1% Pen/Strep (Gibco, 15140-122). and seeded into barcoded, tissue culture microplates (Corning, 3712BC) at ~8000 cells per well (cpw) using a MultiFlo FX multimode dispenser (Agilent, Santa Clara, CA, USA). The cell microplates were incubated overnight at 37 °C, 5% CO_2_, ≥90% relative humidity (RH) with coverage by a standard plate lid (Supplementary Materials and Methods S1). Infection medium was prepared by spiking plant medium with 1 µM Shield-1 ligand (Merck & Co., Inc., Rahway, NJ, USA or ClonTech, Vancouver, BC, Canada, 632189). Immediately prior to assay, samples were thawed at ambient temperature and only lyophilized samples reconstituted in 0.9% saline solution (TEKnova, Hollister, CA, USA, P1391) or ultrapure water (Invitrogen, 10977-015). The reference standard, positive control, and test samples were first prediluted as needed in infection media. Samples were then titrated 2-fold in infection media across ten wells of a 384-well low-attachment plate (Thermo Scientific, 4309) (Figures S1 and S2). The diluted samples were transferred to the ARPE-19 cell plates and incubated for 20 h at 37 °C, 5% CO_2_, ≥90% RH to allow the virus to infect cells.

### 2.4. Immunostaining and Imaging

Infected ARPE-19 cells were fixed with 3.7% formaldehyde (Sigma, St. Louis, MO, USA, F1635) in 1× phosphate-buffered saline (PBS) for 30 min, permeabilized with 0.5% Triton X-100 (Sigma, T8787) in 1× PBS for 20 min, and blocked with 1.0% bovine serum albumin in 1× PBS (TekNova, P1391) for 30 min. The cells were then incubated at ambient temperature with an anti-IE1 monoclonal antibody (mouse anti-human anti-IE1, clone L-14) (Sino Biological, Beijing, China) for 1 h followed by an anti-species fluorophore conjugate (chicken anti-mouse Alexa Fluor^®^ 488 IgG H+L) (Life Technologies, A21200) for 30 min to label HCMV-expressing nuclei and Hoechst 33342 (Life Technologies, Carlsbad, CA, USA, H3570) for 5 min to label nucleic acids for total cell enumeration (Supplementary Materials and Methods S2). After each staining step, cells were washed three times with 1.0% Tween-20 (Sigma Aldrich, P9416) in 1× PBS. The microplates were imaged using a fluorescence imager (BioTek Cytation3) at 4× magnification at two fields of view (FOVs) in two fluorescent channels, DAPI and GFP (Figure S4A, Supplementary Materials and Methods S2).

### 2.5. Assay Automation

A custom, automated system was designed to perform the cell-based analytical methods involved in the IRVE assay, capable of screening up to 44 plates permitting the testing of 264 samples using a 96-well plate format or 528 samples using a 384-well plate format (Figure S3). The system was contained within an enclosure (HighRes Biosolutions, Beverly, MA, USA) outfitted with HEPA filtration, maintained at negative pressure, with BSL-2 certification and vented externally to the building. A protocol replicating the established IRVE method was developed in Cellario software (HighRes Biosolutions, version 3.5.1.78) to perform all required sample and cell plate processing steps, including serial dilution, infection, incubation, fixation, staining, and imaging (Supplementary Materials and Methods S3).

### 2.6. Data Analysis

Using predefined parameters, the Gen5 software (Agilent, version 3.09) enumerated the total number of cells based on Hoechst 33342-positive nuclei and infected cells based on IE1-positive nuclei in each well (Figure S4B). The percent infected cells metric was calculated using

% infected cells = (number of infected cells)/(number of total cells) × 100(1)

and the data extracted. Custom Visual Basic-coded Excel workbooks were used to graph the percent infected cells per well against the dilution factor to generate four-parameter logistic (4PL) curve fits for the HCMV reference standard, PC and test samples (Figure S2). Based on the dilution curves, a half-maximal effective dilution (ED_50_) value was calculated representing the dilution at which 50% of the cells in the well were infected. The ED_50_ value for the PC and each test sample was compared to the ED_50_ of the reference standard to determine the percent relative potency according to

% rP = (reference ED_50_)/(sample ED_50_) × 100 × dilution factor. (2)

Infectious unit (IU) titers for bridging the IRVE assay to the plaque assay were calculated according to

Titer (IU/mL) = −ln *P(0)* × *n*/*v* where *P(0)* = (*n* − number of infected cells)/*n* (3)

where *P(0)* = (*n* − number of infected cells)/*n*, *n* = number of cells in each well at the time of infection and *v* = undiluted virus input volume [26].

### 2.7. Statistics

Statistical significance was evaluated using a one-way ANOVA or *t*-test in GraphPad Prism (Graphpad, Boston, MA, USA, version 10.2.2397) with a *p*-value ≤ 0.05 considered statistically significant. Variance Component Analysis (VCA) was performed using a nested ANOVA in Minitab (Minitab, State College, PA, USA, version 22.1.0) assuming a 95% confidence interval to calculate the coefficient of variation (CV). Pearson r correlations were performed in GraphPad Prism (Graphpad, version 10.2.2397) by applying a transform and simple linear regression to the data.

## 3. Results

### 3.1. Cell Density and Serum Optimization

**Figure 1:**
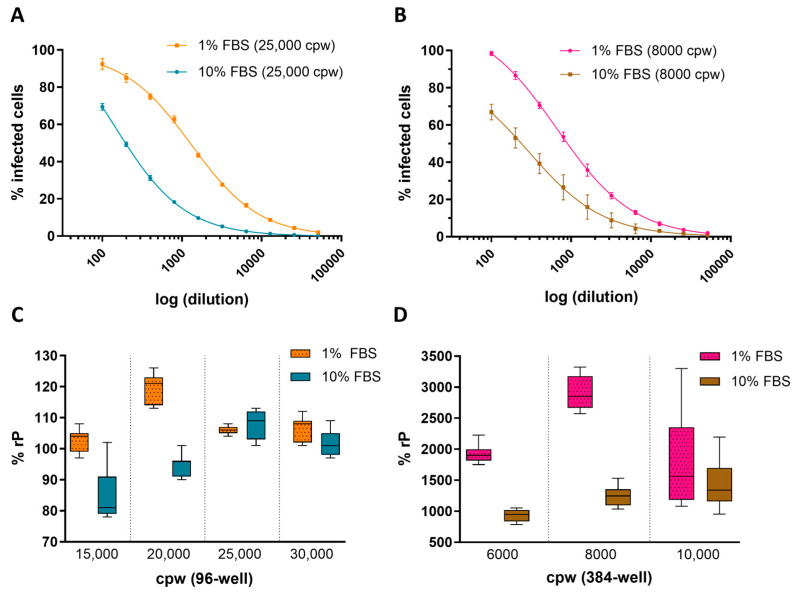
Impact of cell density and serum concentration on HCMV infection. Representative 4PL-fit dose–response curves show the average percentage of cells infected (% Infected Cells) by HCMV DS measured in a (**A**) 96-well plate seeded 25,000 cpw in 1% or 10% FBS (*N* = 8 replicates per dilution point) and (**B**) 384-well plate seeded at 8000 cpw in 1% FBS (*N* = 11 replicates per dilution point) or 10% FBS (*N* = 14–16 replicates per dilution point). Dose curve error bars represent the standard deviation (SD) from the mean. The % rP of (**C**) a HCMV DS sample was measured in 96-well plates seeded at 15,000–30,000 cpw in medium containing 1% or 10% FBS (*N* = 7 replicates) and (**D**) a different DS sample tested in 384-well plates seeded at 6000–10,000 cpw in medium containing 1% or 10% FBS (*N* = 11–12 replicates). Box plots display the median and SD across *N* = 7 replicate dose–response curves per condition.

Key assay parameters, including cell culture density and FBS concentration in the cell media, were evaluated across 96- and 384-well plate formats to establish optimal conditions for ARPE-19 HCMV infection in the IRVE assay. Figure 1A,B shows the impact of FBS concentration in cell infection media on the percent infected cpw with a decrease in the number of infected cells at 10% FBS compared to 1% FBS, regardless of well plate format. Furthermore, Figure 1C,D demonstrates the impact of 10% FBS on relative potency at different seeding densities, with lower densities showing a decrease in potency (96-well: 15,000 and 20,000 cpw; 384-well: 6000 and 8000 cpw) and higher densities showing a lesser effect (96-well: 25,000 and 30,000 cpw; 384-well: 10,000 cpw). Overall, the 96- and 384-well conditions resulting in the highest potency utilized infection media containing 1% FBS and plates seeded at 20,000 and 8000 cpw, respectively.

**Figure 2:**
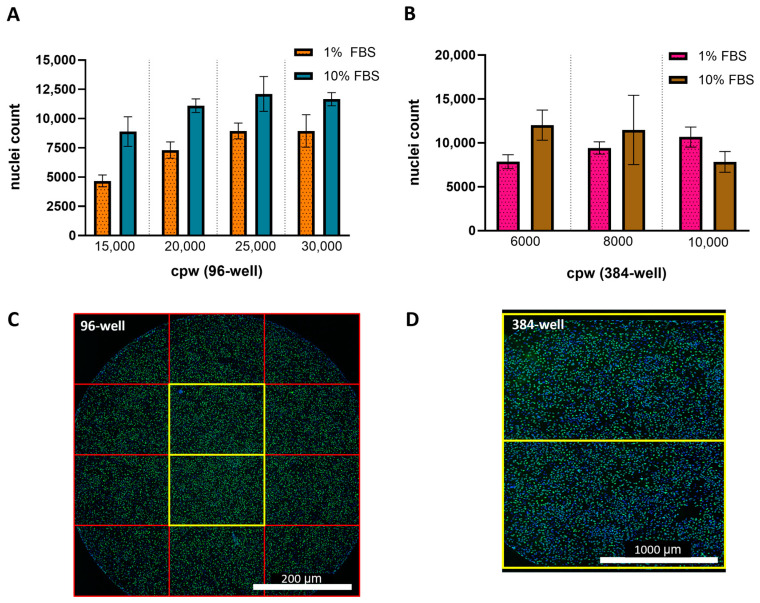
Impact of serum concentration and cell seeding density on nuclei counts after infection. The average Hoechst-positive nuclei count after infection in 1% FBS (unshaded bars) or 10% FBS (shaded with diagonal lines) for a (**A**) 96-well plate (*N* = 96 wells across one plate) and (**B**) 384-well plate (*N* = 384 wells across one plate). Error bars represent the SD from the mean. Stitched montage of a (**C**) 96-well or (**D**) 384-well. Each square represents a single FOV with yellow squares marking the images collected for cellular analysis. Total surface area of a 96-well is 32 mm^2^ which requires 12 FOVs to cover >90% of well, where the 2 center FOVs cover ~20% of the well. Total surface area of a 384-well is 7 mm^2^ with 2 FOVs covering >80% of the well.

In addition to the percent of infected cells and relative potency measurements, cell counts based on nuclei detection were measured in response to changes in FBS concentration and seeding density. Figure 2A,B shows that for all seeding densities and plate formats tested, the 10% FBS resulted in higher cell counts Figure 2C,D displays representative fluorescence microscopy images of 96- and 384-wells containing HCMV infected ARPE-19 cells and the FOVs used for quantitation. In general, the 96- and 384-well conditions resulting in the lowest variability used infection media containing 1% FBS and seeded at 15,000 and 8000 cpw, respectively. All assay characterization studies going forward were collected in a 384-well plate format using infection media containing 1% FBS with cells seeded at 6000 or 8000 cpw, since these conditions provided the highest throughput and lowest variability.

### 3.2. Infection Time and Cell Age Evaluation

**Figure 3:**
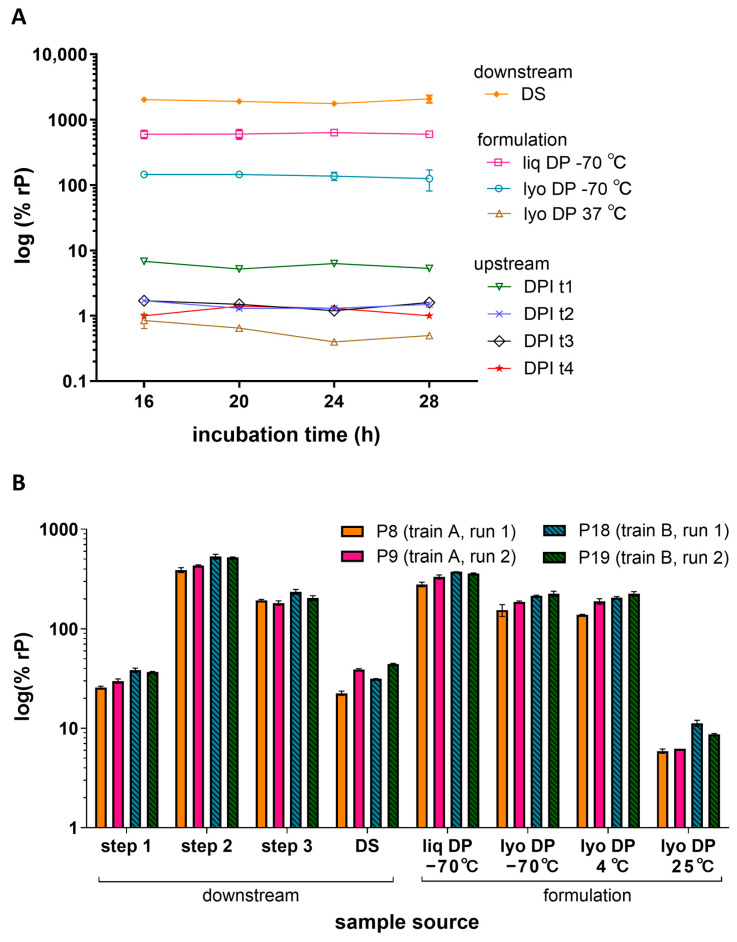
Infection time and cell age. (**A**) Relative potencies for various HCMV vaccine processes and formulation samples were measured after different infection incubation times (16, 20, 24, or 28 h) in 384-well plates seeded with ARPE-19 cells at a density of 6000 cpw. The plot shows the % rP (log10) of upstream process samples across four different day post-infection (DPI) timepoints t1-t4 (*N* = 1), downstream process DS sample (N = 8) and formulated DP samples as liq or lyo with the lyo sample stored at a low (−70 °C) or high (37 °C) temperature (*N* = 2). Error bars represent SD from the mean for samples with *N* ≥ 2. (**B**) Relative potencies for downstream process and formulation samples were measured across different cell passage numbers (low: P8 and P9; mid: P18 and P19), cell trains (train A, train B), and runs (run 1, run 2). Different purification steps (Steps 1, 2, and 3), the DS, and liq and lyo DS samples after storage at different temperatures (−70 °C, 25 °C). Samples were tested in ARPE-19 cells seeded at a density of 8000 cpw in 384-well plates. Error bars represent the value range between *N* = 2 replicates.

To characterize the impact of infection time and cell age on the measured relative potency, various post-inoculation incubation times and cell passage (P) numbers were evaluated. In one study, ARPE-19 cells were inoculated with various HCMV vaccine process samples and allowed to incubate for 16, 20, 24, or 28 h (Figure 3A). The infection incubation timepoints evaluated displayed generally low variability (<20% CV) in measured potency for samples of >1% rP. The lyophilized DP stored at 37 °C showed the highest variability across timepoints and the lowest potency. Overall, deviations in incubation times ranging from 16 to 28 h did not have a drastic impact on potency results.

To determine the impact of ARPE-19 cell attributes such as aging, cells of different passage numbers and trains were evaluated across multiple runs. Figure 3B displays representative relative potency results for various downstream process and formulation samples across two assay runs (run 1 and 2) using two different cell trains (train A and B) at low-range (P8 and P9) and mid-range (P18 and P19) passage numbers. The mean relative potency per sample measured within the same passage, train, and run had 0–14% CVs whereas the mean relative potency measured across different passages, trains, and runs had a slightly increased variance of 12–29% CVs depending on the sample type. A relatively greater potency was observed at higher passage for lyophilized DP samples stored at 25 °C; however, the mean relative potency for most samples was not greatly impacted by a differing cell train or passage number ranging from P8 to P19. Additional passage studies were performed at higher passages (P24 and P31) which showed no significant difference between P8 and P24 (all *p*-values >0.05, *N* = 12 samples). However, a 1.5–2.5-fold increase in potency was measured between P18 and P31 (all *p*-values < 0.05, *N* = 12 samples) (Figure S5). Based on these studies, infection times of 16–28 h and cell age of P8–P24 showed little impact on measured relative potency in the IRVE assay.

### 3.3. Variance Component Analysis

**Figure 4:**
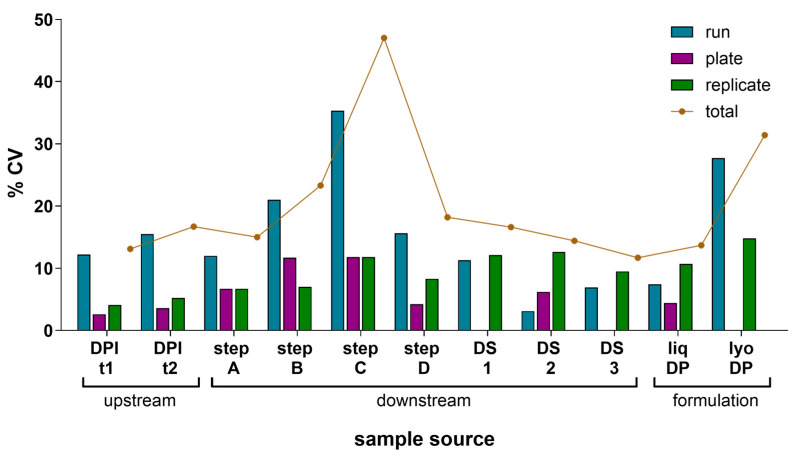
Component variance analysis of relative potency measured. The component variance of mean relative potency in the IRVE assay was calculated for 11 different HCMV vaccine samples with each sample tested across *N* = 3 assay runs with each run occurring on a different day, and each sample tested on *N* = 3 plates per run at *N* = 4 replicates per plate. For each sample type, the plots show the mean response as % rP and the CV in % rP among three components (runs, plates, and replicates) and the total CV. Samples were acquired from upstream at two different days post-infection (DPI) time points (t), downstream for four different intermediate steps (step A–D) and three DS samples (DS 1–3), and formulation as liquid (liq) or lyophilized (lyo) DPs. CVs of zero represent samples with no measured variance.

A precision study was performed to evaluate the IRVE assay’s total variance and the source of this variance under the established assay conditions informed by Section 3.1 and Section 3.2 results. To assess IRVE assay precision, various HCMV vaccine development samples were evaluated, and a VCA was performed on the results (Figure 4 and Table S1). Each sample was tested in four true replicates per plate (replicate-replicate) for three replicate plates (plate-plate) across three assay runs (run-run) over three days for a total of 36 analyses per sample. In this nested study design, a total variance of <20% CV was measured for most of the sample types. A total variance > 20% CV was observed for step B, step C, and lyophilized DP samples with rationale justified in Section 4.2.5. Overall, the variance contributed by the plate and replicate components was small, ranging from 0 to 12% and 4–15% CV, respectively, while most of the total variance was attributed to the run component which ranged from 3 to 35% CV. A separate study was conducted to evaluate the contribution of vial-vial variability (Results and Discussion S1).

### 3.4. Correlation to IEE and Plaque Assays

**Figure 5:**
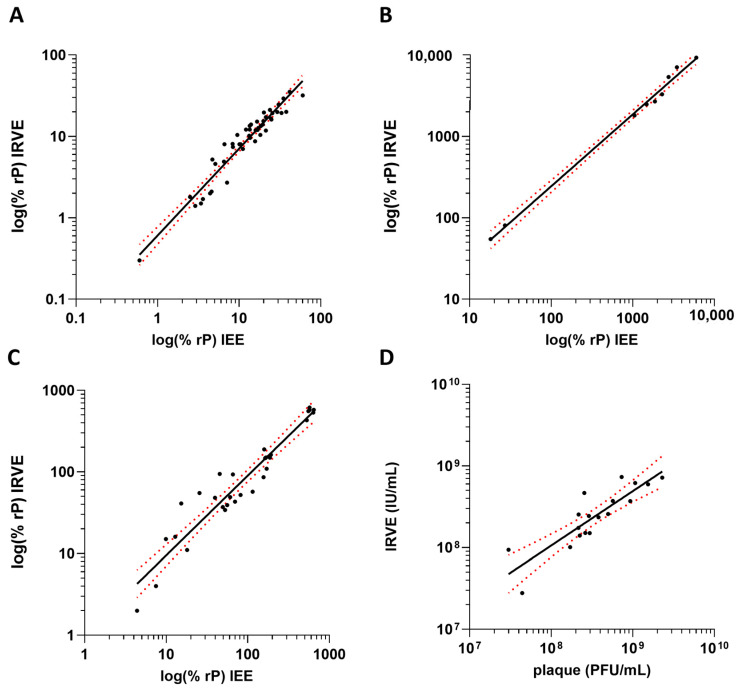
Correlation of IRVE to IEE and plaque. The correlation plots compare IRVE to IEE and plaque assay potency results. IRVE bridging to IEE is displayed for samples from (**A**) upstream process where y = 1.118x − 0.30 and r = 0.98 (*p* < 0.001) (*N* = 74 samples), (**B**) downstream process where y = 0.88x + 0.62 and r = 0.98 (*p* < 0.001) (*N* = 7 samples) and (**C**) formulations where y = 0.98x + 0.0021 and r = 0.95 (*p* < 0.001) (*N* = 30 samples). (**D**) IRVE correlation to plaque where titers reported for IRVE and plaque are reported in Infectious Units (IU) and Plaque Forming Units (PFU) where y = 1.13x − 0.97 and r = 0.87 (*N* = 18 samples). For all plots, the black solid line represents the transform and simple linear regression, and the red dotted line represents the 95% confidence interval.

The 384-well IRVE assay was bridged to its predecessor infectivity assay the 96-well Immediate Early Gene Expression (IEE) assay using the same anti-IE1 antibody [24] and the classical 6-well plaque potency assay (Supplementary Materials and Methods S4 and S5). For the bridging of IRVE to IEE, HCMV vaccine development samples were tested in both assays and a correlation was applied to the relative potency data (Figure 5A–C). The IRVE and IEE assay performance displayed a strong correlation for each sample type tested, with Pearson r > 0.95 and *p* < 0.001. For upstream samples, a similar trend was observed across both assays with potency increasing with increasing viral propagation time. Variability was similar between the two assays, generally coming in between 0 and 20% CV. Similarly, bridging of IRVE to the classical plaque potency assay was accomplished by testing various HCMV vaccine development samples across both assays and the final calculated potencies plotted for correlation (Figure 5D). The infectious unit concentration and plaque forming unit potency results from the IRVE and plaque assay, respectively, displayed a correlation of Pearson r = 0.87.

### 3.5. Vaccine Development Case Studies

**Figure 6:**
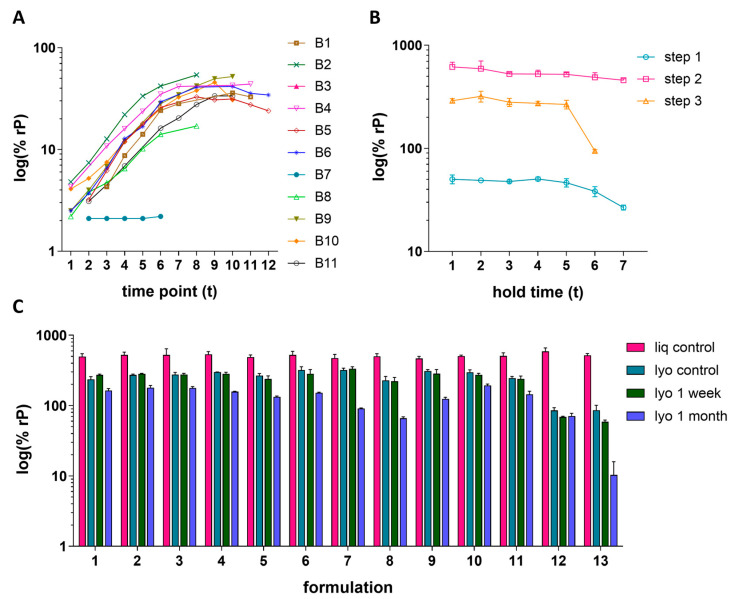
Routine application of IRVE assay to HCMV vaccine development. (**A**) Upstream process: Bioreactors (B1–B11) were processed under different culture conditions. Throughout viral propagation in ARPE-19 cells, 5–11 time point samples were collected for each bioreactor in singlet (94 total samples). The plot displays the %rP (log10) at each time point. The average PC response in the assay run was 47.3% ± 2.8% rP (*N* = 36 total plates) and the results shown were acquired from plates 1–8. (**B**) Downstream process: Three different process intermediate steps were evaluated. Samples were collected and put on hold at ambient temperature for stability testing after each step with three replicate vials collected for each hold time point (63 total samples). Error bars represent the SD from the mean (N = 3 vials). Average PC response was 41.9% ± 4.0% rP (*N* = 19 total plates) and the results shown were acquired from plates 9–15. (**C**) Formulation: A total of 13 different formulations were prepared as liq or lyo DPs and stored for 1 week or 1 month (*N* = 52 sample types). Three replicate vials were prepared for each of the 52 sample types (*N* = 156 total samples). Displayed is the average % rP (log10) for each sample type where error bars represent the SD from the mean (*N* = 3 vials). The average PC response across all plates in the run was 45.4% ± 3.0% rP (*N* = 27 total plates) with the results shown acquired from plates 8–27.

Three case studies are presented to demonstrate the IRVE assay applications to the vaccine upstream process, downstream process, and formulation development studies. In the upstream study, HCMV vaccine construct was propagated in ARPE-19 cells across 11 different bioreactor (B) conditions and 12 time points (t) (Figure 6A). The bioreactor study included conditions evaluating the effects of iterative serial expansions of ARPE-19 cells on microcarriers in combination with a shift in the targeted timing of infection of the culture. Generally, an increase in relative potency was measured as time progressed with a plateau starting around t8–t9. The negligible potency of B6 was directly correlated to an unintended bioreactor event causing abnormally high pH which led to inhibition of viral propagation. Of the bioreactors operating as intended, B2 and B8 displayed the highest and lowest overall potency throughout the time course, respectively. A loss of potency at later time points was observed for some conditions, with B10 showing the greatest potency loss after t10. Overall, two of the leading bioreactor conditions, B2 and B4, demonstrated both high and consistent potency over time.

The downstream case study shows how three different, sequential processing steps impacted HCMVs relative potency, as well as the stability of the samples when held at ambient temperature across seven time points (Figure 6B). This study demonstrated an increase in potency from step 1 to step 2, as expected due to the concentration of the sample by capturing chromatography and tangential flow filtration [25]. Between Step 2 and Step 3, the batch is further purified and experiences dilution, as reflected by the potency decrease. Generally, a decrease in potency was observed when holding the processed samples at ambient temperature for long periods of time, with the step 3-processed sample showing drastic stability loss from t5–t6. This study highlights a potency risk for the Step 3 intermediate. It can be hypothesized that the impurities for the less pure samples (Step 1 and Step 2) provide a stabilizing effect for the process stream.

In the formulation case study, the relative potency of 13 different HCMV vaccine formulation samples each formulated with different excipient combinations were prepared as liquid or lyophilized cakes and stored under different conditions (Figure 6C). Storage conditions include immediate storage at −70 °C or storage at 4 °C for 1 week or 1 month. In general, results showed a maintenance of potency across the different liquid formulations and loss upon lyophilization or storage at higher temperatures and for longer periods of time. The preservation of potency after lyophilization (i.e., lyophilized yield) and log loss of potency due to storage at 4 °C can be further visualized in Figure S6. Formulations 12 and 13 lead to the lowest lyophilization yield with 13 also displaying the largest log loss after 1 month at 4 °C. Formulations 7 and 9 showed the highest lyophilization yield; however, both displayed greater loss in potency at 4 °C compared to other formulations. Ultimately, formulations displaying a relatively more ideal balance between higher lyophilization yield and lower log loss, including 1–6, 10, and 11, served as rational for further exploration.

### 3.6. Assay Performance Monitoring

**Figure 7:**
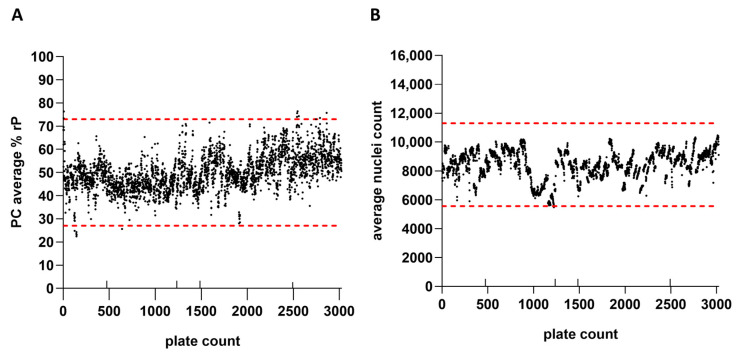
IRVE assay performance was monitored across thousands of well plates. Results are displayed for 3022 384-well plates tested across 131 assay runs, 13 scientists, and 2 automated systems. (**A**) rP of the PC sample per plate with the lower and upper acceptance limits displayed as red dashed horizonal lines at 27% and 73%, respectively. (**B**) Average nuclei count per plate with the lower and upper acceptance limits displayed as red dashed horizontal lines at 5555 and 11,309, respectively. The additional up-facing notches on the x-axis represent a change in reference standard (plate 472 and 1234) and PC lot (plate 1485), as well as implementation of alternative commercial reagents and instrumentation for assay modernization (plate 2493). Assay acceptance limits were established according to a three-sigma calculation (average ± SD × 3).

IRVE assay performance was monitored across thousands of microwell plates. The plate performance parameters monitored for each run included the PC relative potency, average nuclei count, and reference ED_50_. Figure 7 shows the average PC relative potencies and nuclei counts for 3022 384-well cell plates evaluated across 131 assay runs and 13 scientists. Based on average relative potency and nuclei count per plate data, changes in reference and PC lots, as well as use of alternative reagents and instrumentation, did not appear to drastically impact assay performance. The average relative potency for the PC and average well nuclei counts per plate inherently fluctuated between upper and lower acceptance limits over several years. Upper and lower plate-based assay acceptance limits for each of the performance measures were defined based on plate performance across a predefined number of runs. Notable fluctuations in performance across plates were attributed to intended method modifications or unintended protocol deviations. Results near and outside of the assay acceptance limits would initiate further investigation and potential retesting of samples (~4% retest rate).

## 4. Discussion

### 4.1. Cell-Based Assay Scaling Considerations (96-Well vs. 384-Well)

Cell-based potency assays are frequently performed in 96-well or lower-well density plates for vaccine process development support. Increasing the well density to 384-well or higher enables increased sample testing capacity and more in-depth screening of process conditions [27]. The IRVE assay was developed across both 96- and 384-well plates for scale comparison and method optimization. The sample layout within a 96-well plate (8 rows − 12 columns) allowed for the testing of eight samples in singlet, with each sample corresponding to a different row (rows A–H) and the samples serially diluted across the plate from columns 2–11 (Figure S1). The sample layout within a 384-well plate (16 rows × 24 columns) permitted the testing of 14 samples in duplicate, whereby each sample corresponded to a different row (rows B–O) and the samples serially diluted across the plate from column 2 to 11 (replicate 1) and column 14 to 23 (replicate 2), respectively (Figures S1 and S2). Each plate format also incorporated wells containing only infection medium, predominately at the plate boarders, to lessen evaporation from sample wells during incubation. Two sample positions in each plate were occupied by a reference and positive control sample, making the final sample capacity six samples per 96-well plate and 12 samples per 384-well plate. The 384-well plate layout permitted a two-fold increase in sample capacity and additional replicate testing for increased precision and confidence while allowing for outlier detection from procedural or automation errors.

Potential concerns with miniaturizing cell-based assays include the lower number of cells evaluated, confinement of cells to a smaller volume and fewer cell–cell interactions forcing the cells into a less physiological state [27,28]. For characterization and development of a pre-clinical vaccine candidate, in vitro potency assay miniaturization from 96- to 384-well may lead to further deviation from in vivo infection kinetics. In efforts to match the infection kinetics observed in the 96- and 384-well plates, a key point of focus was to have a cell monolayer of high confluency (~100% cell coverage across the well bottom) in the plates at the time of viral infection. The imaging methods for 96- and 384-well plates were also aligned to collect images from two center well locations using the same objective so that a similar total number of cells was measured. Regardless of plate format, the ED_50_ values shifted when the number of cells per well or FBS concentration was changed (Figure 1 and Figure 2). However, the relative sample potencies calculated for each plate format were similar.

Development of the IRVE assay across plates of lower and higher well densities has extended the assay applicability to laboratories not equipped with the expertise or equipment required to process higher-density plates. With comparability demonstrated between the assay formats, the 384-well plate assay was progressed further through development for more thorough characterization and to pair with automation to facilitate higher throughput vaccine candidate sample testing.

### 4.2. Minimizing Sources of Cell-Based Potency Assay Variability

It is essential to characterize sources of variability and optimize key parameters during assay development to improve the reproducibility of data. Cell-based assays particularly experience high variability in results due to method complexity and inherent heterogeneity of biological systems. Assays utilizing cells as a substrate tend to involve numerous steps occurring over several days to weeks, increasing the likelihood of variation between assay runs [28,29]. Additionally, single-cell heterogeneity is always present and evolving due to a combination of intrinsic and extrinsic factors regardless of culture clonality. Cell culture conditions can significantly affect cell population dynamics, including growth rate, health, gene expression, and sensitivity to viral infection [30,31,32]. To reduce variability in the IRVE assay where possible, parameters suspected to be key variables effecting potency measurements were evaluated for their impact on assay precision. The conditions identified as most favorable were applied to the established method. The parameters screened included the cell seeding density, infection media serum concentration, infection incubation time, and cell age. Instrumentation used for cell seeding into well plates and methods for sealing plates during incubation were also evaluated to minimize well-well variability in cell count and evaporation during incubation (see Supplemental Results).

#### 4.2.1. ARPE-19 Cell Density Alters HCMV Infectability

Cell culture density directly impacts cell–cell interactions which can modulate various factors including susceptibility to viral infection [33]. Studies have also demonstrated that the epithelial ARPE-19 cell line displays the expected epithelial cell behavior as a fully confluent monolayer but can also display mesenchymal-like behavior when grown as a subconfluent monolayer [34]. To encourage consistency in ARPE-19 infectability and cell behavior, the number of cells seeded per well were selected so that, compared to other conditions, the relative potency was high, variability across replicates was low and a near 100% confluent monolayer was achieved prior to infection (Figure 1 and Figure 2). Under this guidance, 25,000 and 8000 cpw were selected for seeding 96- and 384-well plates, respectively. Interestingly, when the infection media FBS concentration was kept constant, potency did not consistently trend with changes in cell count, demonstrating the complex cellular dynamics involved in viral infectivity.

#### 4.2.2. FBS Inhibits HCMV Infection in ARPE-19 Cells

FBS is an animal serum containing a large amount of nutrients commonly added to the cell culture medium to promote in vitro cell growth [35]. However, FBS composition is complex and the impact on gene regulatory pathways in cells can vary with batch and brand [36]. It is crucial to identify FBS conditions that provide sufficient assay response and repeatability. Low (1% FBS *v*/*v*) and high (10% FBS *v*/*v*) concentrations were evaluated in the IRVE assay, with the 10% FBS mostly resulting in decreased HCMV infection rate, increased variability in measured potency between replicates, left-shifted ED_50_ indicating decreased sensitivity and faster ARPE-19 cell growth (Figure 1 and Figure 2). The impact of different FBS batches (e.g., lots) was not independently evaluated. However, assay ruggedness as measured by the PC’s relative potency and average nuclei count was demonstrated over time whereby different consumables lots were used (Section 3.6 and Section 4.2.5, Figure 7).

A decrease in the HCMV infection rate at the higher FBS concentration likely results in the cells becoming more resistant to infection. Several studies have suggested that FBS and similar sera can contain factors that block or promote viral infection in a dose-dependent manner by modifying the attachment of certain viruses to target cells or by modulation of the target cell’s gene expression [37,38,39,40]. Additionally, since FBS promotes in vitro cell growth, higher serum level cultures are likely to have higher cell counts which can alter cell sensitivity to infection as discussed previously.

Increased assay variability at 10% FBS likely results from the generally faster cell growth, since the change in cell count from the start to end of infection would be larger and potentially more variable over time. The 1% FBS condition generally slowed down cell growth allowing for more precise control of cell count at time of infection. However, there were instances of similar and lower cell counts achieved upon seeding 8000 and 10,000 cells, respectively, in 384-well plates in 10% FBS-containing media. This divergence speaks to the nature of cell-based research and suggests a shift in cell biology resulting from the more confined 384-well or other parameters not evaluated in this study.

The dose–response curves at 10% FBS were missing an upper asymptote at the sample concentrations tested, whereas the 1% FBS condition provided a complete sigmoidal curve with both lower and upper asymptotes. To implement 10% FBS in the infection medium, higher HCMV sample concentrations would be required to try to achieve the upper asymptote. However, higher-concentration samples may not be readily available or cause matrix interference in the assay. To reduce variability and increase sensitivity of the IRVE assay, the 1% FBS concentration was selected to supplement the cell infection medium.

#### 4.2.3. IRVE Demonstrates Robustness Across Different Infection Times

The time permitted for a virus to infect the target cells prior to cell fixation and infection measurement can skew potency assay results. Too short of an infection time can lower measured signal, precision, and dynamic range of the assay. Too long of an incubation may cause extensive viral propagation and cell death; however, due to HCMVs slow replication cycle (four to five days), this point was not a concern. To achieve robust sample potency results, it is valuable to establish the optimal infection time based on assay readout requirements and characterize any variance caused by shifts in incubation time to establish the acceptable range. For HCMV infection in ARPE-19 cells, previous reports showed that a 20 h infection incubation permitted sufficient IE1 expression for quantitation by immunostaining [41]. An incubation time of 20 h would also accommodate typical workday hours for scientists running the assay manually.

Automation of the IRVE assay permitted the screening of additional timepoints outside of workday hours, including 16, 24, and 28 h, to characterize the impact of infection time on relative potency measured. Downstream and formulated DP samples stored at −70 °C remained relatively unaffected by the change in infection times across 16–28 h (Figure 3A). Opposingly, samples with relatively lower potency, including upstream and DPs stored at 37 °C, did show some variation in potency across timepoints. However, the potency shifts did not trend with infection time suggesting that variability was intrinsic to the assay when testing low-potency samples.

In later studies, a relatively earlier time point of 8 h, and later timepoints of 32 h and 36 h were also tested. The 8 h condition permitted adequate signal for quantitation and the 32 h and 36 h timepoints showed potential increases in measurement variability. The robustness of the IRVE assay at 8, 32, and 36 h, however, has not yet been thoroughly characterized and would require further study. Based on the evaluation results acquired, the IRVE assay was routinely operated with a 20 h infection incubation time, with a 16–28 h window of operability.

#### 4.2.4. Limiting ARPE-19 Cell Age Improves Assay Repeatability

Cell passage history is foundational to the overall response measured in a cell-based assay. Changes in gene regulation can be induced by changes to the cell’s microenvironment and can also result from more natural causes such as aging. Cell age, usually quantified by cell passage number, can alter sensitivity and response to viral infection [42]. ARPE-19 cells are known to undergo gene expression changes and epithelial–mesenchymal transition with increased passage under certain culture conditions [43]. Therefore, the impact of passage number (low: P8/P9, mid: P18/P19, high: P24/P31) on relative potency was evaluated in the IRVE assay to establish an upper passage limit for the ARPE-19 master cell bank. Two different cell trains (i.e., different cell vials from the same master cell bank) were also evaluated to characterize the variance resulting from different vials of cells.

The relative potencies measured across the different ARPE-19 cell trains, passage number, and runs were generally repeatable, with the variance measured as ≤29% CV (Figure 3B), with the total variance compounded by different sample runs, plates, and replicates measured as ≤31% CV (Figure 4 and Table S1). Because most of the variance in the IRVE assay is attributed to the run variable, variance observed under different cell train and passage number conditions may also be largely compounded by run-run variance. A greater potency was observed at higher passage for lyophilized DP samples stored above 4 °C for one sample set (Figure 3B), but not the other (Figure S5). The difference in potency then is suspected to result less from passage number and more so from decreased sample stability under specific temperatures and formulation conditions which can lead to increased variability in potency.

Across the ARPE-19 passages evaluated, consistency in relative potency measured within known assay variability was established for P8, one passage after vial thaw at P7, through P24. Additional testing of cells between P24 and P31 would be required to determine the precise age at which potency changes significantly. In the IRVE assay, the ARPE-19 cells were not used past P24 to ensure consistency of cell response to infection. The set limits permitted each cell train to be used for 16 passages at a once-per-week interval, thus typically 4 months prior to ending the culture. If a new ARPE-19 master cell bank is ever generated, the cell age study should be repeated to re-establish the passage limit.

#### 4.2.5. IRVE Variance Is Dependent on HCMV Vaccine Sample Type

Relative potency variance resulting from key assay operating conditions was first characterized for the IRVE assay (Section 3.1 and Section 3.2). With the final assay conditions established and the assay automated, the variance due to less controllable sources, including sample plate, replicate, run, and vial components were quantified across different HCMV vaccine sample types (Figure 4, Table S1, Supplementary Results). Characterization of the IRVE assay’s intrinsic variability was critical to understanding the statistical significance of the relative potencies measured and reporting meaningful results for making process decisions during vaccine development.

In the VCA evaluation, a fresh aliquot of each sample was thawed immediately prior to testing in each run to avoid any potential freeze–thaw and stability effects on potency. Therefore, run-to-run variability results will also encompass any vial-to-vial variability present within a sample which can contribute to the higher run-to-run variance observed. The total variance of the IRVE assay was generally <20% CV for most HCMV vaccine process sample types, with a few exceptions. Higher assay variability was likely observed due to the instability and/or low potency of the sample. For example, downstream process intermediates step B and step C contained relatively high salt concentrations compared to other process intermediates, causing a loss of sample stability during sample freeze–thaw and then, lower relative potencies (4.3% rP step B; 0.9% rP step A) and higher total assay variance (23% CV step B; 47% CV step C). Additionally, the lyophilized DP displayed a higher total variance (31% CV) compared to the liquid DP, likely due to altered sample stability with lyophilization and higher temperature storage. Vial-vial variability caused by lyophilization procedures and manual reconstitution of lyophilized cakes prior to the assay may also contribute to the higher variability (Supplementary Results). The VCA evaluation provided insight into the statistical significance of relative potency assay results when making process and formulation decisions during vaccine development, with <20% CVs expected for most sample types, but >20% CVs possible for less stable or low potency samples.

### 4.3. IRVE Correlates to IEE and Plaque

The previously established HCMV potency assays, IEE and plaque, have been successfully used to support HCMV vaccine clinical release and stability testing. However, IEE and plaque assays could not accommodate the higher sample counts and faster turnaround time for data required to streamline the vaccine process and formulation optimization efforts during early-stage development. Although the IRVE assay successfully addresses previous throughput limitations, it requires specialized instrumentation for automation which are not readily available in most laboratories and manual processing of 384-well plates can be challenging. Thus, the IRVE assay was not meant to replace IEE or plaque during later-stage clinical release and stability testing, though it was critical to determine whether the results were predictive of those vaccine potencies. Assay bridging studies showed a strong correlation of IRVE to IEE and plaque, demonstrating that IRVE could be used to effectively predict IEE and plaque assay results (Figure 5).

When comparing the two relative potency assays, IRVE and IEE, potencies were generally lower by IRVE for upstream and formulation samples, but higher for downstream samples. Magnitude differences between the two assays may result from various factors including the different reference standards, signal acquisition method (cell count vs. well intensity), plate format (384-well vs. 96-well), and liquid handling (automated vs. manual) (Supplemental Methods). The sample type can also alter variability in assay response, as noted in Section 4.2.5. When plotting the IRVE and IEE data for correlation, deviations in slope for samples from different stages of vaccine development were observed; therefore, the correlation plots were prepared and analyzed separately for each sample type.

The correlation of IRVE to plaque could not be performed separately for each sample type due to the limited number of replicates available. The relatively weaker correlation measured for IRVE to plaque, compared to IEE, likely results from the fewer number of replicates and drastic method differences including plate format (384-well vs. 6-well), incubation time (16–28 h vs. >20 days), signal acquisition (cell count vs. plaque count) and protein measured (IE1 vs. glycoprotein B) (Supplemental Methods). Replicates were partially limited by the low throughput of the plaque assay. Additionally, only IRVE results associated with <25% infected cells were analyzed to ensure most cells were infected only by a single virus, since the calculated titer can be underestimated when >30–40% cells are infected due to viral spread [26]. Additional data would be required to further explore the differences observed between sample types and assays; however, the correlations are promising and informative for process development decisions.

### 4.4. Established IRVE Assay Demonstrates Ruggedness

The IRVE assay has been successfully and routinely utilized to evaluate the potency of various HCMV vaccine samples originating from different stages of the vaccine development pipeline, including the upstream process, downstream process, and formulation of DP (Section 3.5 and Figure 6). Assay performance monitoring over several years and across thousands of microwell plates enabled thorough characterization of the assay’s ruggedness in response to method modifications, including changes in reference and/or PC lot, consumable lot and/or manufacturer, instrumentation model and/or vendor, and operating scientist (Section 3.6 and Figure 7). To minimize fluctuations in assay results caused by procedural changes, the new and old procedures were compared and bridged across multiple runs of the assay. For example, when implementing a new reference lot, the reference sample’s dilution was ultimately adjusted to elicit a similar assay response (e.g., percent cells infected) as the previous lot used. As another example, when a different anti-species fluorophore conjugate lot was used which displayed intensity differences, the IE1 image intensity threshold segmentation was adjusted to match the previous. Assay bridging acceptance criteria were based on the VCA study, with bridging considered successful when the variance between and within procedures was at or below the expected variance of the assay (Section 3.3 and Section 4.2.5; Figure 4; Table S1). The extended application IRVE assay to support HCMV vaccine development demonstrates the assays impressive ruggedness in response to often inevitable changes to consumables, technology, and other laboratory processes.

## 5. Conclusions

The IRVE assay was successfully developed and applied as a high-throughput screening tool for the process and formulation development of a live-attenuated HCMV vaccine candidate. Valuable findings include the inhibition of ARPE-19 cell infectability by HCMV in media comprising a high FBS concentration and specific cell densities in 96- and 384-well plates. Results demonstrated minimal impact of HCMV infection time on relative potency and more notable shifts in relative potency when infecting high passage number cells. IRVE consistently measured changes in relative potency across different vaccine processes and formulation conditions with a strong correlation to manual IEE and plaque potency assay results and routinely showed increased assay variability when testing samples of lower stability and potency as anticipated. The IRVE platform showcases the benefits of automated potency assays, enabling more in-depth vaccine development studies, decreasing labor burden on scientists and improving assay robustness and ruggedness as demonstrated across thousands of sample microplates. Future work may involve the evolution of IRVE methods to enable more clinically relevant characterization of HCMV mechanism of action underlying entry, replication, and lytic activity.

## Data Availability

The data presented in this manuscript are available on request from the corresponding author. The data are not publicly available due to the proprietary nature of the work which was conducted in compliance with requirements of the current legal framework at MSD. Data pseudo-anonymized are however available from the MRL labs upon reasonable request to any researcher wishing to use them for non-commercial purposes and will have to be approved by Merck & Co., Inc., Rahway, NJ, USA legal.

## Supplementary Materials

The following supporting information can be downloaded at https://www.mdpi.com/article/10.3390/vaccines13060626/s1, Figure S1: 96-well and 384-plate layouts for IRVE assay; Figure S2: Correspondence of the 384-well plate format and resulting dilution curves; Figure S3: Automated IRVE Assay Platform; Figure S4: Cellular imaging and analysis for measuring HCMV infection; Figure S5. Impact of high passage number ARPE-19 cells on HCMV infection; Figure S6. Additional Analyses for Formulation Application Study; Table S1: Component variance analysis of mean response in the IRVE assay; Supplementary Materials and Methods S1: Plate Sealing Methods; Supplementary Materials and Methods S2: IRVE Imaging Method Development; Supplementary Materials and Methods S3: IRVE Imaging Parameter Selection. Supplementary Materials and Methods S4: IRVE Assay Automated Workflow; Supplementary Materials and Methods S5: IEE Assay; Supplementary Materials and Methods S6: Plaque Assay; Results and Discussion S1: Formulation Vial Variance Study.

## Abbreviations

The following abbreviations are used in this manuscript:

HCMV	Human cytomegalovirus
ANOVA	Analysis of variance
ARPE	Arising retinal pigment epithelia
ATCC	American type culture collection
Chrom	Chromatography
CO2	Carbon dioxide
CV	CV
Cpw	Cells per well
DAPI	4′,6-diamidino-2-phenylindole
DMEM/F-12	Dulbecco’s Modification of Eagle’s Medium and Ham’s F-12 nutrient
DNA	Deoxyribonucleic acid
DS	Drug substance
DP	Drug product
DPI	Days post-infection
ED50	Half maximal effective dilution
FBS	Fetal bovine serum
FOV	Field of view
GFP	Green fluorescent protein
gH	Glycoprotein H
h	Hour
HCMV	Human cytomegalovirus
IE1	Immediate Early 1
IU	Infectious unit
IRVE	Imaging of Relative Viral Expression
IEE	Infectivity of Early Gene Expression
Liq	Liquid
Lyo	Lyophilized
Min	Minute
P	Passage
PBS	Phosphate-buffered saline
PC	Positive control
Pen/Strep	Penicillin/Streptomycin
PFU	Plaque forming unit
RH	Relative humidity
rP	Relative potency
SD	Standard deviation
t	Time point
TCID50	Tissue culture infectious dose 50
VCA	Variance component analysis
*v*/*v*	Volume to volume ratio
Wk	Week
