# Supplementary material for: A High-Throughput and Robust Relative Potency Assay Measuring Human Cytomegalovirus Infection in Epithelial Cells for Vaccine Development
Source: Vaccines (Basel). 2025 Jun 10;13(6):626. doi: 10.3390/vaccines13060626 (PMC12197749; doi:10.3390/vaccines13060626)
Supplement: Supplementary file 1 [file vaccines-13-00626-s001.zip › vaccines-3618897-supplementary.pdf]

A high-throughput and robust relative potency assay measuring human cytomegalovirus infection in epithelial cells for vaccine development

Nicole M. Smiddy [1,*], Nisarg Patel [1,*,†], Matthew C. Troutman [1,‡], Kristine M. Kearns [1,§], Zachary P. Davis [1,‖],
Christopher S. Adams [1], Carl Hofmann [1], Donald J. Warakomski [1], Harrison Davis [2], Daniel Spatafore [3], Adam Kristopeit [3], Pete DePhillips [1] and John W. Loughney [1]

1 Analytical Research and Development, Merck & Co., Inc., Merck & Co., Inc., 770 Sumneytown Pike, West Point, PA 19486, USA, USA; matt.troutman83@gmail.com (M.C.T.); kristine.kearns@optimizeyourlab.com (K.M.K.); zpdavis93@gmail.com (Z.P.D.); chris.adams1@merck.com (C.S.A.); carl.hofmann@merck.com (C.H.); donald_warakomski@merck.com (D.J.W.); pdsld@comcast.net (P.D.); john_loughney@merck.com (J.W.L.)

2 Vaccine Drug Product Development, Merck & Co., Inc., Merck & Co., Inc., 770 Sumneytown Pike, West Point, PA 19486, USA, USA; harrison_davis@merck.com

3 Process Research and Development, Merck & Co., Inc., Merck & Co., Inc., 770 Sumneytown Pike, West Point, PA 19486, USA, USA; daniel.spatafore@merck.com (D.S.); adam_kristopeit@merck.com (A.K.)

* Correspondence: nicole.smiddy@merck.com (N.M.S.); npatel886@live.com (N.P.)

† Current address: Analytical Development, Genentech, 3025 Market Street, Philadelphia, PA 19104, USA.

‡ Current address: High Throughput Screening & Analytics, Interius Biotherapeutics, 3401 Grays Ferry Avenue, Building 200, Suite 300, Philadelphia, PA 19146, USA.

§ Current address: Optimize Laboratory Consultants, LLC., 2217 Locust Dr., Lansdale, PA 19446, USA.

‖ Current address: Analytical Operations, Gilead Sciences, 333 Lakeside Drive, Foster City, CA 94404, USA.

Figures and Tables

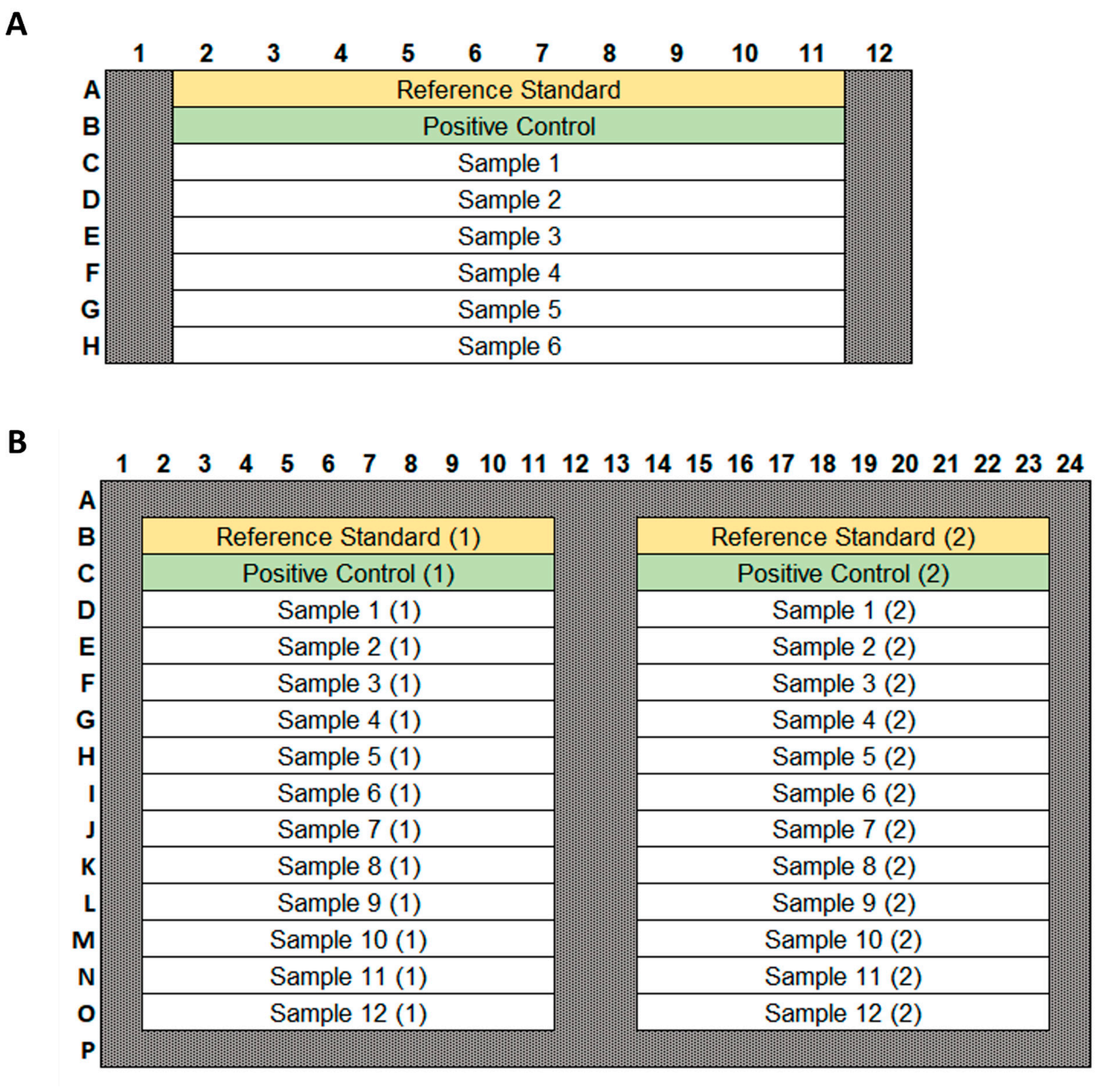

Figure S1. 96-well and 384-plate layouts for IRVE assay. (**A**) The 96-well assay plate layout allows for a total of 8 samples per plate (1 reference standard, 1 positive control, and 6 test samples) which are added to Col 2 (single replicate) and serially diluted across the plate. For assay replicates, an additional plate must be added, or samples can be tested in duplicate row which would permit 3 test samples per plate. Columns 1 and 12 contain media only. (**B**) The 384-well assay plate layout allows for a total of 14 samples per plate (1 reference standard, 1 positive control, and 12 test samples) which are added to Column 2, replicate (1) and Column 14, replicate (2) followed by 2-fold serial dilution across the plate. Columns 1, 12, 13, and 24, as well as rows A and P contain media only.

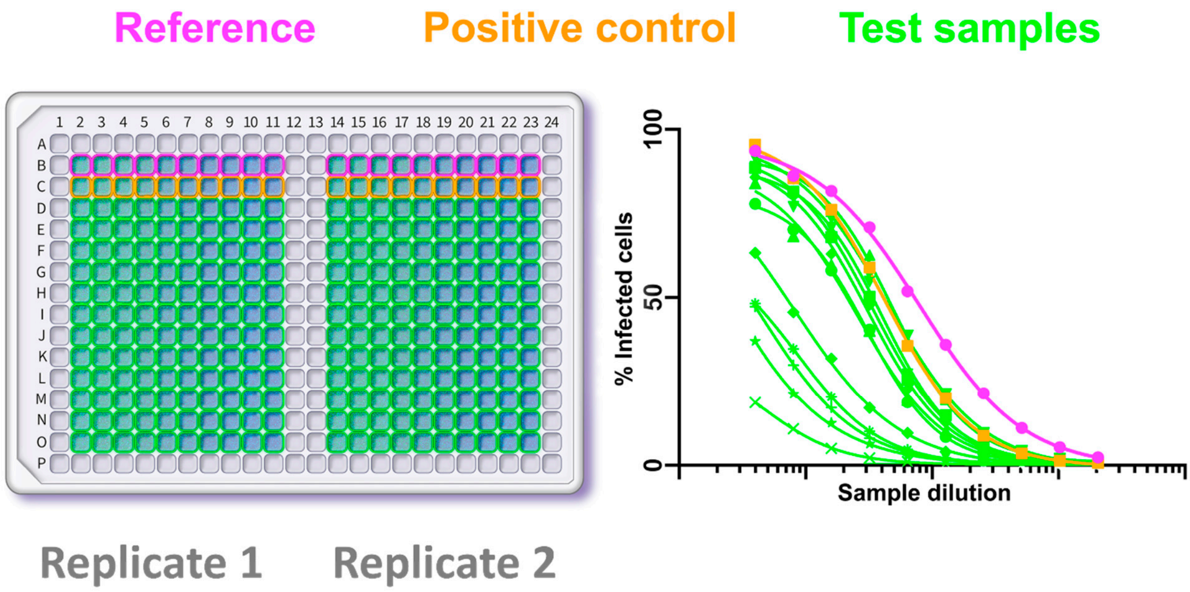

Figure S2. Correspondence of the 384-well plate format and resulting dilution curves. The 384-well plate format permits the testing of 14 samples per plate in duplicate (replicate 1 and replicate 2), including 1 reference (row B), 1 positive control (row C), and 12 test samples (rows D-O). Samples are added to plate column 2 (replicate 1) and column 14 (replicate 2) which are serially diluted 2-fold across the plate for a total of 10 different dilutions. The % infected cells result for each sample at each dilution are calculated and plotted with four-parameter logistic curve fits applied. From the 4PL-fit curves, the ED_{50} of each positive control and test sample is compared to the ED_{50} of the reference to determine the percent relative potency (% rP) for each sample.

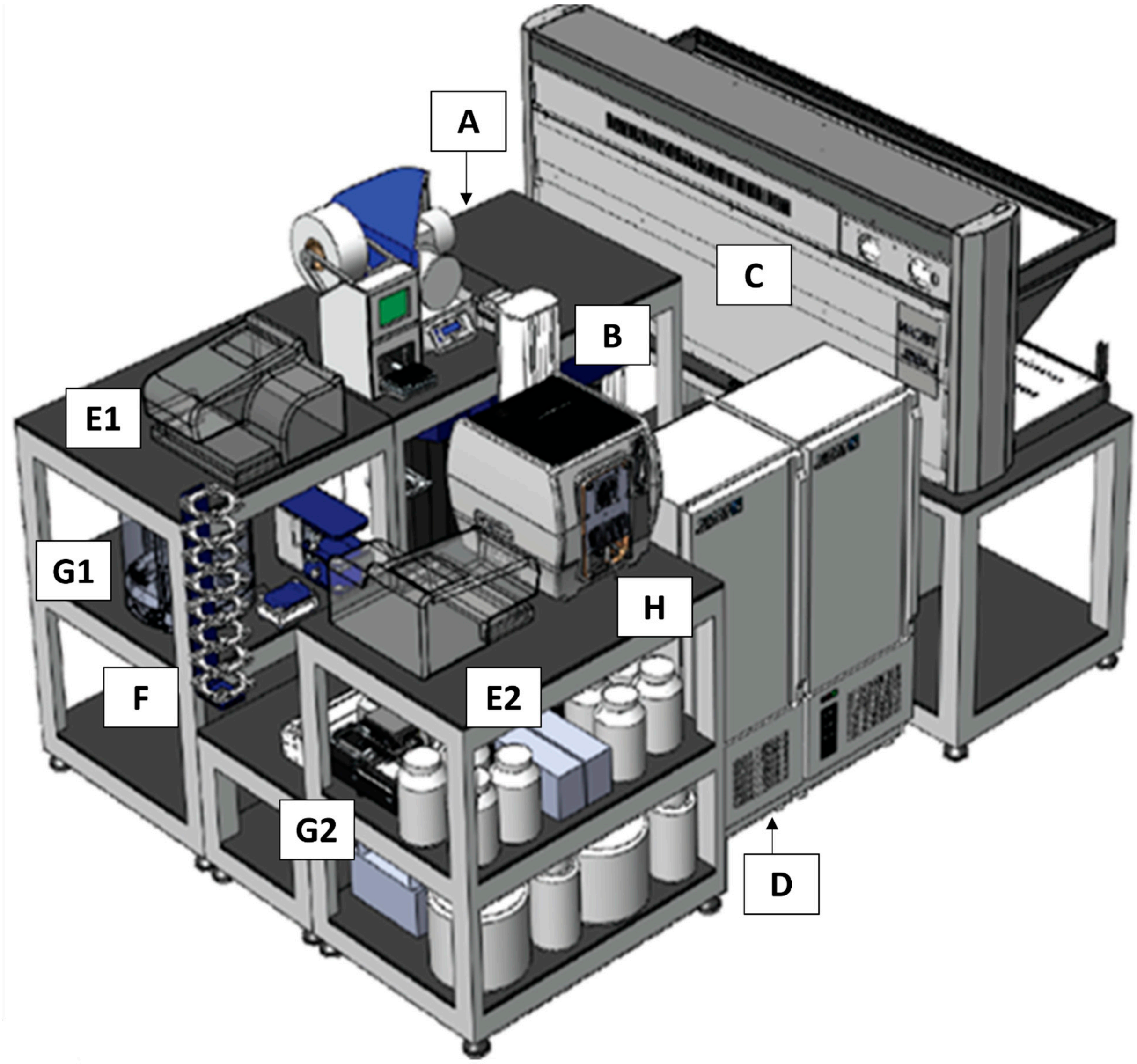

Figure S3. Automated IRVE Assay Platform. Schematic showing location of key components utilized for the IRVE assay. (**A**) Ambistore Carousel for microplate and tip box storage. (**B**) ACell robotic arm that moved along a rail for microplate transfers. (**C**) Freedom EVO 200 liquid handler for sample serial dilutions and cell infection. (**D**) LiCONiC STX 44 ICSA 37 °C CO_2 incubator for infection incubation. (**E_1, E_2**) MultiFlo FX Dispensers for cell fixative and staining reagent addition. (**F**) 8-position Plate Hotel for ambient incubation during cell fixation. (**G_1, G_2**) ELX405 Washers for cell microplate washing and buffer addition. (**H**) Cytation3 imager for cellular imaging and analysis. The entire system was contained within an HRB enclosure (not shown).

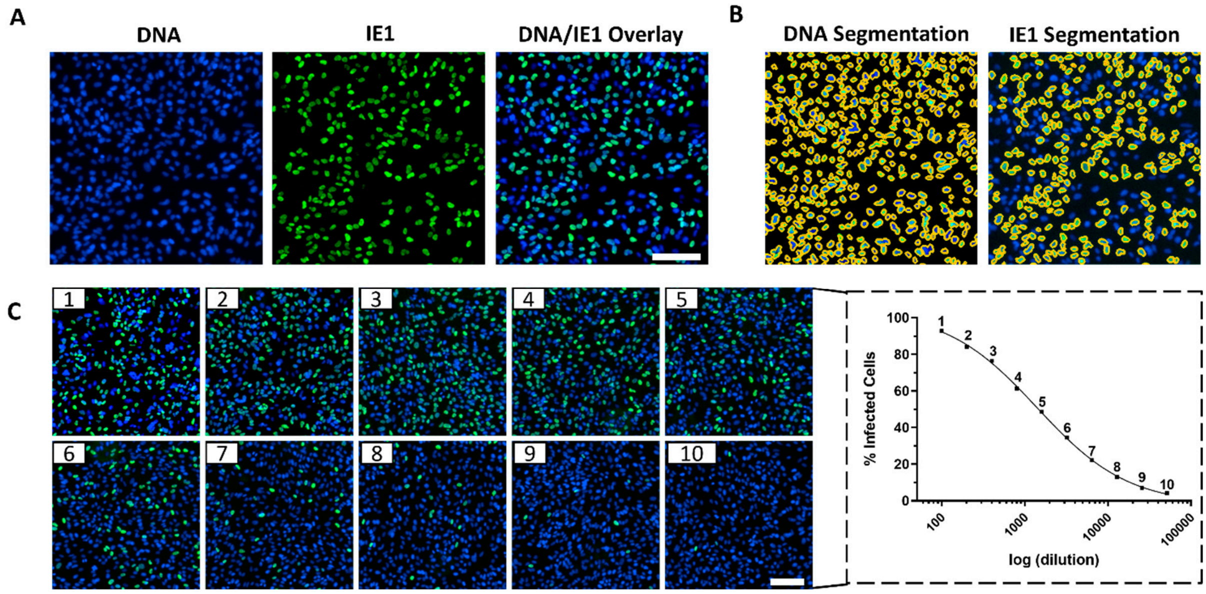

Figure S4. Cellular imaging and analysis for measuring HCMV infection. ARPE-19 cells incubated with HCMV vaccine samples are ultimately stained, imaged, and analyzed to enumerate infected cells per well. Images shown are representative examples of (**A**) cellular nucleic acids marked with Hoechst 33342 (blue, DAPI filter), IE1 protein (green, GFP filter), and an overlay of the nucleic acid and IE1 images showing areas of signal overlap, (**B**) segmentation of nucleic acid-positive and IE1-positive cell nuclei after intensity and size thresholding (yellow boarders) which are used to enumerate total cells (nucleic acid) and infected cells (IE1), and (**C**) Percent infected cells at ten different sample dilutions which are used to generate dose response curves where percent infected cells are plotted against the dilution (displayed as $\log_{10}$). The numbers 1-10 overlayed onto each image correlates with the dilution point on the example dose response curve where 1 and 10 correlates to the lowest and highest dilution, respectively. Scale bars for all images are 200 μm.

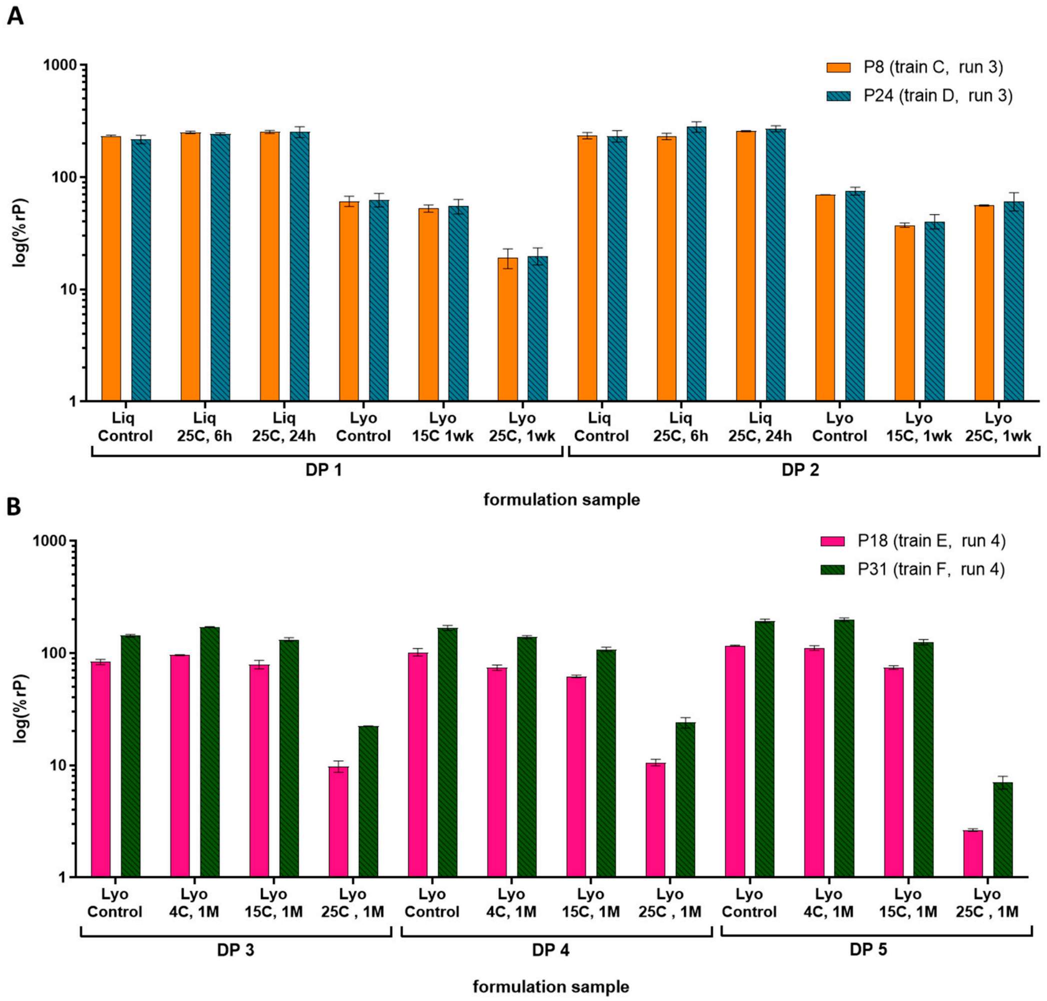

Figure S5. Impact of high passage number ARPE-19 cells on HCMV infection. Relative potencies for HCMV vaccine drug product (DP) samples under different formulations (DP 1-5) were measured across different cell passage numbers (P), cell trains, and assay run dates. Samples were tested in ARPE-19 cells seeded at a density of 8000 cpw in 384-well plates. (A) % rP (log_{10}) of liquid (liq) DP control and DP stored at 25 °C for 6 h or 24 h for time in solution studies, as well as lyophilized (lyo) DP control and DP stored at 15 °C or 5 °C for 1 week (wk) for accelerated stability studies. Samples were tested in two different cell trains (Train C, Train D) at low (P8), or mid (P24) passage number within the same run. (B) % rP (log_{10}) of lyo DP stored at different temperatures (4 °C, 15 °C, 25 °C) for 1 month (M). Samples were tested in two different cell trains (Train E, Train F) at mid (P18) or high (P31) passage number within the same run. Bars on the plots representing low passage number results are not shaded, whereas high passage results are shaded with diagonal lines. Error bars represent the value range between $N = 2$ replicates.

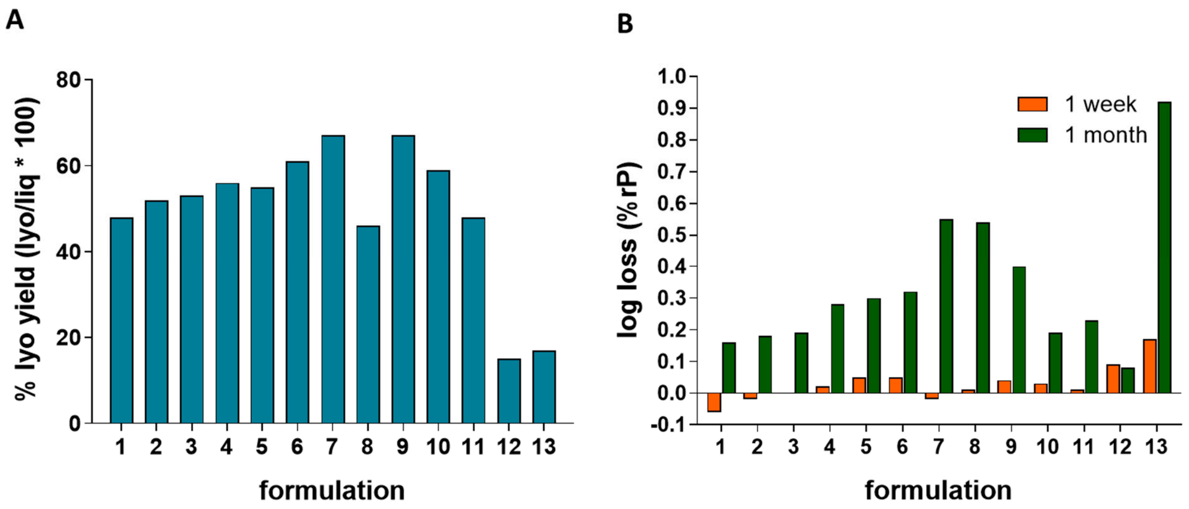

Figure S6. Additional Analyses for Formulation Application Study. IRVE assay results for HCMV vaccine formulations prepared as liquid (liq) or lyophilized (lyo) DP as reported in **Figure 6C**, were further analyzed to quantitate lyo yield and log loss. (**A**) The average % rP yield (average lyo % rP/ average liq % rP × 100) with a lower yield indicating a decrease in % rP upon lyophilization. (**B**) Average log loss in % rP after storage at 4°C for 1 week or 1 month, as compared to the lyo control sample immediately stored at -70 °C, was calculated according to log(average lyo control % rP) – log(average lyo 4°C % rP). PC response was 45.4% ± 3.0% rP (N = 27 total plates in assay run) with the results shown here acquired from plates 8-27.

Vaccine Process	hCMV Sample	Mean Response (% rP)	Run-Run Variance (% CV)	Plate-Plate Variance (% CV)	Rep-Rep Variance (% CV)	Total Variance (% CV)
Upstream	DPI t1	58.1	12.2	2.6	4.1	13.1
	DPI t2	50.2	15.5	3.6	5.2	16.7
Downstream	chrom A	32.6	12	6.7	6.7	15
	chrom B	4.3	21	11.7	7	23.3
	chrom C	0.9	35.3	11.8	11.8	47
	chrom D	167.2	15.6	4.2	8.3	18.2
	DS 1	4316.0	11.3	0	12.1	16.6
	DS 2	4387.0	3.1	6.2	12.6	14.4
	DS 3	4526.0	6.9	0	9.5	11.7
Formulation	liq DP	1371.0	7.4	4.4	10.7	13.7
	lyo DP	497.8	27.7	0	14.8	31.4

Table S1. Component variance analysis of mean response in the IRVE assay. The component variance of mean response in the IRVE assay was calculated for 11 different HCMV vaccine samples with each sample tested *across* $N = 3$ assay runs with each run occurring on a different day, and each sample tested on $N = 3$

plates per run at $N = 4$ replicates per plate. For each sample type, the plots shows the mean response as % rP and the component variance in % rP as CV among three components (runs, plates, and replicates) and the total CV. Samples were acquired from upstream at two different day post-infection (DPI) time points (t), downstream after purification through four different chromatography columns (Chrom A-D) three DSs (1-3), and formulated liq and lyo DPs. CVs of zero represent samples with no measured variance.

Materials and Methods

Supplementary Materials and Methods S1: Plate Sealing Methods

A standard well plate lid and gas-permeable seal were evaluated (N=1 384-well plate per condition) for use during the IRVE assay's infection incubation step, which took place for 20 ± 4 h at 37 °C, 5% CO_2, 90% relative humidity following viral sample addition to the cell plate. It was discovered that the plate with the gas-permeable seal experienced increased evaporation and a relative potency loss of 63% when compared to the lidded plate. The gas-permeable seal also resulted in a loss of sensitivity with the ED_{50} on the infection curve shifting left by a factor of 2.7 compared to the lidded plate ED_{50}. Thus, covering of the well plate with a lid during the infection incubation step was found to be superior to sealing as it resulted in less evaporation and offered better sensitivity.

Supplementary Materials and Methods S2: IRVE Imaging Method Development

The relative potency (rP) of HCMV vaccine samples was measured in the IRVE assay using fluorescence microscopy and single-cell image analysis, as demonstrated in **Figure S4**. Specifically, ARPE-19 cells were incubated with HCMV samples to allow for cell infection, immunofluorescently stained for intracellular IE1 protein expressed during early HCMV infection and additionally stained for nucleic acids. Fluorescence images were collected at each serial dilution, cell segmentation algorithms were used to segment the nuclei of single cells, and the number of total and infected cells counted by setting fluorescence intensity and nuclear size thresholds. Representative fluorescence images shown demonstrate sufficient nuclear signal of IE1 protein and nucleic acid above background for quantitation. The decreased number of infected cells with increased HCMV sample dilution can also be clearly observed across all ten

HCMV concentration for a single sample both visually in the images and quantitatively in the dose response curve.

Cell plate imaging parameters, specifically the number of FOVs per well collected and analyzed on the Cytation3 Imager, were evaluated for 96-well and 384-well formats. A 96-well plate required 12 FOVs to capture >95% of the well at 4× magnification. To reduce imaging time and data storage burden, assay results for the 12 FOVs compared to a subset of the well (<12 FOVs) were evaluated. It was determined that the analysis of 2 FOVs (18% of the well) was sufficient to provide adequate representation of the entire well. A 384-well plate requires two FOVs to capture >80% of the well at 4× magnification. Although fewer cells are seeded in a 384-well, there is improved image representation of the entire well overall.

Supplementary Materials and Methods S3: IRVE Assay Automated Workflow

Beginning with the cell plating step, automation was utilized for the final assay procedure. The cells were plated with using a BioTek MultiFlo FX and BioStack plate module (Agilent, Santa Clara, CA, USA). The cell plates were incubated in an integrated incubator (LiCONiC, North Reading, MA, USA) overnight. The next day, the reference standard, positive control, and test samples were pre-diluted and reformatted into the proper plate positions using an offline Tecan Freedom EVO 150 (Tecan, Männedorf Switzerland). The sample plate was moved to the integrated system and placed onto the integrated Tecan Freedom EVO 200 for serial dilutions. The ACell robotic arm (HighRes Biosolutions, Beverly, MA, USA) moves the cell plate from the LiCONiC incubator onto the Tecan. The samples are stamped from the serial dilution plate onto the cell plate which is then returned to the incubator. Following the overnight incubation, the plates were transferred from the incubator to the ELX405 washers (Agilent) where the infection media is removed, and 3.7% formaldehyde is added to each plate. After the cells are fixed, the washers remove the formaldehyde and add 1× PBS to the plates until they are ready for immunostaining. The immunostaining process consisted of a series dispense and washing steps. The washing steps were performed by the ELX405 washers which aspirate the liquid and add a wash buffer to the plates. The dispense steps, which were performed by the MultiFlo FX dispensers, included permeabilization, blocking, primary

antibody, secondary antibody, and Hoechst 33342 stain in sequential order. After immunostaining, the plates remained at ambient temperature in the Ambistore Carousel (HighRes Biosolutions) until they were moved to the Cytation 3 plate reader (Agilent) for imaging and analysis.

Supplementary Materials and Methods S4: IEE Assay

The Immediate Early Gene Expression (IEE) assay was conceptualized using previously reported HCMV neutralization assay methods [1]. The IEE assay was routinely utilized for HCMV vaccine development prior to IRVE assay development [2]. ARPE-19 cells (ATCC, Manassas, VA, USA, CRL-2302) were seeded in planting media at 2.5 × 10^5 cpw in 96-well plates and incubated at 37 °C and 5% CO_2 for 24 ± 4 h at 37 °C and 5% CO_2. Infection medium was prepared by spiking plant medium with 3 μM Shield-1 ligand (Merck & Co., Inc., Rahway, NJ, USA). The HCMV reference standard, PC, and test samples were pre-diluted as needed followed by serial dilution in infection media across a 96-well low-attachment U-bottom plate (Corning, Corning, NY, USA, 3799). The diluted samples were transferred to the ARPE-19 cell plates and the virus allowed to infect the cells for 24 ± 4 h at 37 °C and 5% CO_2. After infection, the cells were fixed with 3.7% Formaldehyde (Polysciences, Inc., Warrington, PA, USA, 04018) in 1× PBS for 25 ± 5 min and permeabilized with 0.1% Triton-X (Sigma, St. Louis, MO, USA, T6284) in 1× PBS (Merck & Co., Inc., Rahway, NJ, USA) for 5 min. The cells were incubated with anti-IE1 monoclonal antibody (Sino Biological, Mouse a-human IE mAb, clone L-14, HB8554) in 0.2% Tween 20 (Sigma, P1379) in Odyssey blocking buffer (Li-COR, Lincoln, NE, USA, 927-40,000) at 2-8 °C for 14-96 h or ambient (18-28 °C) for 2-6 h. After primary staining, cells were incubated with an anti-species fluorophore conjugate (Goat a-mouse IgG (H+L) IR Dye 800CW) (Li-Cor, 926-32210) for 2-6 h at ambient temperature. After each staining step, cells were washed three times with wash buffer comprised of 1× PBS and 0.05% Tween 20(Merck & Co., Inc., Rahway, NJ, USA). The plates were air-dried before scanning at 800 λ and a resolution of 200 using a LI-COR SA Odyssey Aerius Imaging System (LI-COR Biotech). A curve fit was calculated for 800 λ intensities across dilution points for each reference standard, PC and test

sample. Test sample and PC ED_{50} values were compared to the reference ED_{50} values to determine their relative potency (% rP).

Supplementary Materials and Methods S5: Plaque Assay

ARPE-19 cells were seeded in maintenance media (DMEM/F12, 1% FBS, 1% Amphotericin, 1% Penn Strep) at 2 $\times 10^6$ cpw in 6-well plates and incubated at 37 °C and 5% CO_2 for two days. Samples were serially diluted, and each dilution was added to a 6-well plate well. The plates were incubated for 2 h at 37 °C and 5% CO_2, the media removed, and maintenance media containing 0.5% Agarose (Lonza, Basel, Switzerland, 50101) added. The plates were incubated at 4 °C for 15 min to allow the agar to solidify and then returned to the incubator for ≥20 days to allow for plaque formation. A sterile spatula was used to remove the overlay media, and 100% methanol (Sigma, 676780) was added to each well for 10 min followed by three washes with 1× PBS. The cells were immunostained at ambient temperature by blocking with milk (Research Products International, Mount Prospect, IL, USA, M17200) for 1h, incubating with primary antibody (Rabbit anti-glycoprotein B 272.7) (GenScript, Piscataway, NJ, USA) for 1 h followed by a secondary antibody (Rabbit IgG) (Vector Laboratories Inc., Newark, CA, USA, PK-6101) for 1 h. After each antibody incubation, the plates were washed three times. The plaques were counted manually under a light box and these values were used to calculate PFU per mL using the equation: $PFU = plaque\ count \times dilution\ factor \times 10$.

Results and Discussion

Supplementary Results and Discussion S1: Formulation Vial Variance Study

A study was carried out to understand the presence of vial-vial variance for formulation samples. Two different formulations (formulation 1 and 2) were tested under liquid control and lyophilized control conditions for a total of four samples. Four replicate vials of each sample (vial-vial) were individually diluted and tested in duplicate per plate (replicate-replicate) over triplicate plates (plate-plate). Assay variability control samples, where the vial-vial variance component was removed, were prepared by pooling the remaining volume from sample vials which were tested in duplicate per plate (replicate-replicate) across triplicate plates (plate-

plate). All conditions for a given sample were tested across the same three plate replicates with varying sample positions to account for positional and plate bias.

For formulation 1, the vial-vial variance for the liquid and lyophilized sample was 6% and 11% CV, respectively, which is within assay variability measured in the earlier VCA study (**Section 3.5**). For formulation 2, the vial-vial variance for the liquid and lyophilized sample was 61% CV and 39% CV, respectively, which was higher than the expected assay variability. Differenced in vial-vial variance depending on formulation showcases the importance of replicate testing for DP samples. Compared to the assay variance control samples, formulation 1 liquid and lyophilized samples were 0.4% and 42.2% different, indicating an increase in vial-vial variability with lyophilization. A similar comparison could not be made from formulation 2 due to the high vial-vial variance.

The higher vial-vial variance for lyophilized samples can be explained by the confounding variance of the lyophilization procedure and manual reconstitution of the lyophilized sample prior to assay. Higher variability for lyophilized samples, especially at higher temperature storage conditions, has been demonstrated in **Figures 3, 4, 6, and S5** and described in **Sections 3.3 and 4.2.5**. Overall, this study demonstrates the importance of formulation vial replicate testing and that the IRVE assay is sufficiently precise to measure small changes in infectivity of formulation samples.

References

1. Tang, A.; Li, F.; Freed, D.C.; Finnefrock, A.C.; Casimiro, D.R.; Wang, D.; Fu, T.M. A Novel High-Throughput Neutralization Assay for Supporting Clinical Evaluations of Human Cytomegalovirus Vaccines. *Vaccine* **2011**, *29*, 8350–8356, doi:10.1016/J.VACCINE.2011.08.086.
2. Spatafore, D.; Warakomski, D.; Hofmann, C.; Christanti, S.; Wagner, J.M. Investigation into the Use of Gamma Irradiated Cytodex-1 Microcarriers to Produce a Human Cytomegalovirus (HCMV) Vaccine Candidate in Epithelial Cells. *J. Biotechnol.* **2023**, *365*, 62–71, doi:10.1016/j.jbiotec.2023.02.005.
